# Biochar Amendment Reduces the Availability of Pb in the Soil and Its Uptake in Lettuce

**DOI:** 10.3390/toxics9100268

**Published:** 2021-10-15

**Authors:** Andrea Vannini, Elisabetta Bianchi, Diego Avi, Nicole Damaggio, Luigi Antonello Di Lella, Francesco Nannoni, Giuseppe Protano, Stefano Loppi

**Affiliations:** 1 Department of Life Sciences, University of Siena, 53100 Siena, Italy; andrea.vannini@unisi.it (A.V.); elisabetta.bianchi@unisi.it (E.B.); diego.avi@student.unisi.it (D.A.); nicole.damaggio@student.unisi.it (N.D.); 2 Department of Physical, Earth and Environmental Sciences, University of Siena, 53100 Siena, Italy; luigi.dilella@unisi.it (L.A.D.L.); nannoni@unisi.it (F.N.); giuseppe.protano@unisi.it (G.P.); 3 BAT Center—Interuniversity Center for Studies on Bioinspired Agro-Environmental Technology, University of Naples ‘Federico II’, 80138 Napoli, Italy

**Keywords:** lead, contaminated soils, biochar amendment, hydroponics, bioavailability

## Abstract

The aim of this study was to investigate the ability of biochar amendment to reduce the availability of Pb in the soil and its uptake in lettuce (*Lactuca sativa* L. var. *adela*). Seedlings of lettuce were cultivated in Pb-contaminated soils, both with and without 5% biochar (*w*/*w*), as well as in a simplified soilless system (hydroponics) at the ecologically relevant Pb concentration of 100 µM, both with and without 1% biochar. Soils amended with biochar resulted in a ca. 50% reduction of the extractable (bioavailable) fraction of Pb, limiting the accumulation of this toxic element in the leaves of lettuce by ca. 50%. A similar behavior was observed for lettuce plants grown hydroponically, even with a much higher reduction of Pb uptake (ca. 80%). Increased cation exchange capacity and pH were likely the main factors limiting the bioavailability of Pb in the soil. Complexation with functional groups and precipitation/co-precipitation both on the biochar surface and in soil aggregates were likely the main mechanisms immobilizing this element.

## 1. Introduction

The contamination of agricultural land with potentially toxic elements (PTEs) is a global concern, as their translocation into food intended for human consumption can pose a potential risk to human health. Among PTEs, cadmium (Cd), copper (Cu) and lead (Pb) have been suggested as requiring strict monitoring in agricultural areas [1]. Lead is one of the most toxic elements for humans and is the cause of a wide array of adverse effects, especially in the most susceptible categories of people such as children and pregnant women [2]. Although Pb contamination of the surface environment (i.e., air and soil) has dropped after the ban of leaded gasoline [3], lead is still regarded as an important contaminant as it is widely used, e.g., to produce car batteries, pigments, ammunition, cable sheathing, weights, crystal glass and radiation protection and to store corrosive liquids; Pb thus has the potential to contaminate the environment, including agricultural soils [4].

Ingestion has always been considered as an important source of Pb intake to humans [2], and the consumption of crops cultivated in soils contaminated by Pb may pose a toxicological concern [5]. As a matter of fact, strategies for the immobilization of Pb in soils are continuously under careful scientific evaluation. Although phytoremediation by means of plant roots is considered a very promising and cost-effective tool for the removal of Pb from contaminated soils [6], the application of this methodology requires time and does not allow the maintenance of routine cultivation activities.

Biochar is a type of charcoal obtained from the thermochemical conversion of biomass in an oxygen-limited environment at temperatures < 700 °C [7,8]. Biochar is mainly produced as a residue of pyrolytic gasification of plant biomass [9,10], including waste from forest management [11] or agriculture [12]. Although biochar is presently used mainly as a soil amendment due to its capacity to improve soil quality, it is also a promising “green” solution to reduce the bioavailability of PTEs in the soil [13], since biochar can efficiently limit the mobility of PTEs and successfully reduce their uptake by plants [14]. Moreover, there is evidence that biochar can profitably be used also for the remediation of polluted waters [15].

Lettuce (*Lactuca sativa* L.) has been ranked as one of the top 10 horticultural plants produced worldwide [16], and it is the most sold leafy vegetable in the USA [17]. This plant, as a good source of nutrients, vitamins and phenolic compounds, is well-known to provide benefits to human health [18]. However, when cultivated in contaminated soils, lettuce can translocate Pb to its aerial parts, thus generating a potential risk for its consumption [19].

The present study was carried out to test whether biochar amendment reduces the bioavailability of Pb in the soil and in a simplified soilless cultivation system (hydroponics) and its uptake in lettuce. The novelty of the work consists in verifying, in a single comprehensive study, the effectiveness of biochar in reducing the mobility and uptake of Pb.

## 2. Materials and Methods

### 2.1. Plant Collection

Forty seedlings of *Lactuca sativa* (L.) var. *adela* (approximate height of 15 cm), cultured in potting soils, were obtained from a local nursery. In the laboratory, the plants were acclimatized for one week in a climatic chamber at a temperature of 20 ± 1 °C, relative humidity (RH) of 80 ± 2% and photosynthetic photon flux density (PPFD) of 300 μmol/m^2^/s with photoperiod of 12 h. One batch of 20 lettuce plants was grown in soil and the other batch in a simplified soilless system (hydroponics). The experiments lasted 6 weeks and were replicated three times.

### 2.2. Soil Culture

Five surface soil samples (depth 0–30 cm) were randomly collected from a locality of Northern Italy affected in the past by a tetraethyl-lead spill, never reclaimed, and recognized as contaminated site of national concern. In the laboratory, each soil sample was dried at 40 °C in a ventilated oven, manually crushed and sieved at 2 mm. The particle size fraction <2 mm of soil samples was divided into two aliquots, one of which was amended with 5% (*w*/*w* dry weight) biochar (BioDea© Black Silt, particle size <500 µm; Table 1). The amount of biochar to be added (5% *w*/*w*) was selected based on the indications of the producer.

Both soil aliquots, with and without the addition of biochar, were then used to fill 20 pots (10 × 10 × 12 cm, 10 per aliquot) in which 20 previously acclimatized lettuce plants were transplanted. All plants were then grown for six weeks inside the climatic chamber at the conditions specified above. All pots were regularly watered with 50 mL of deionized water every two days. In order to avoid any possible influence by microclimatic conditions (i.e., light and temperature), all pots were randomly rotated every two days.

### 2.3. Hydroponic Culture

The remaining 20 lettuce plants were gently separated from their cultivation soil using deionized water. Each plant was then placed in a plastic jar with 350 mL of Atami© B’Cuzz Hydro nutrient solution (pH 5.65), prepared by diluting 2 mL/L of the nutrient solution A and B in deionized water (Table 2). All jars were closed with a plastic cover with a central hole. To help the plants stay upright, synthetic wool (Sera©) was wrapped around their stems. Samples were then placed for one week inside the climatic chamber at the conditions specified above.

After one week, the plants were randomly divided into two aliquots of 10 units (statistical replicates), each receiving a different treatment: the first one was exposed to 350 mL of nutrient solution which contained Pb at the ecologically relevant concentration of 100 μM [21], while the last one was exposed to 350 mL of nutrient solution in which 100 μM Pb and 1% (*w*/*w*) biochar were added. Lead was provided as nitrate salt (Pb(NO_3_)_2_) dissolved in deionized water. Each nutrient solution was contaminated with Pb by adding 1 mL of a solution containing 11.6 mg/mL of the above-mentioned nitrate salt. All plastic containers were maintained under agitation for 5 h to allow the adsorption of Pb to biochar particles [22]. Then, lettuce plants were transplanted into new experimental plastic jars. The samples were grown for six weeks inside the climatic chamber at the conditions specified above, randomizing their position every two days to avoid any possible influence of microenvironmental conditions.

### 2.4. Physical and Chemical Soil Properties

After six weeks of soil culture, soils with and without the addition of biochar were removed from their pots, dried at 40 °C in a ventilated oven, manually crushed and sieved at 2 mm.

The particle size distribution in soil samples, expressed as the percentage content of the sandy, silty and clayey granulometric fractions, was measured by the hydrometer method [23], which is based on Stoke’s law governing the rate of sedimentation of particles suspended in water.

To determine the pH and cation exchange capacity in soil samples, the soil particle size fraction < 2 mm was treated by manual quartering and mechanical pulverization. The pH was measured in a 1:1 (*w*/*v*) soil/water mixture according to the method 9045/D of the United States Environmental Protection Agency [24]. The effective cation exchange capacity (CEC) was determined applying the method of Hendershot and Duquette [25]: concentrations of Ca, Mg, K, Na and Al were measured in a mixture consisting of 2 g of soil sample and 20 mL of 1 mol/L NH_4_Cl solution.

The cation exchange capacity was also measured in biochar applying the method described for soil samples.

### 2.5. Total and Extractable Pb in the Soil

To determine the total Pb content in soils with and without the addition of biochar, the particle size fraction < 2 mm of each soil sample was homogenized by manual quartering and mechanical pulverization. About 250 mg of powdered soil samples were then solubilized by acid digestion adding the following ultrapure reagents: 2 mL HF, 2 mL HNO_3_ and 1 mL H_2_O_2_ [26]. Acid digestion was performed in Teflon bombs in a Milestone Ethos 900 microwave lab station (Sorisole, BG, Italy).

To assay the Pb concentration in the soil extractable fraction, soil samples were processed using acetic acid (CH_3_COOH) as extractant. The soil extractable fraction of a chemical element sums its amounts in water-soluble constituents (water-soluble fraction), adsorbed by ionic exchange on the surface of constituents such as mainly clay minerals and organic compounds (exchangeable fraction) and included in acid-soluble constituents (mainly carbonates) by precipitation and/or co-precipitation (acid-soluble fraction). The soil extractable fraction removed by an organic acid such as acetic acid is considered as representative of the bioavailable amount of a chemical element in the soil [27,28]. The extraction of the soil extractable fraction was carried out applying step A of the chemical sequential extraction procedure proposed by the Community Bureau of Reference [29], as follows: 40 mL of acetic acid 0.11 M solution was added to 1 g of powdered soil sample; the mixture was stirred for 16 h at room temperature and then centrifuged for 15 min at 3500 rpm. The supernatant was removed, filtered and then used for the analysis of the extractable Pb fraction.

The total and extractable Pb concentrations in soil samples were determined by inductively coupled plasma-mass spectrometry (ICP-MS) using a Perkin Elmer NexION 350 spectrometer (Waltham, MA, USA) and were expressed on a dry weight basis. The analytical quality was evaluated using the following certified standard reference materials: NIST 2709 (San Joaquin Soil) and GBW 07411 (Soil) for total Pb concentrations and SRM 701 (Sediment) for extractable Pb concentrations. The recoveries of Pb were 91.9% and 99.8% for NIST 2709 and GBW 07411, respectively, and 101.2% for SRM 701.

The total Pb content was also determined in biochar following the method applied for soil samples.

### 2.6. Total Pb in Lettuce

Lettuce plants were harvested and dried at 60 °C for 2 days, then finely crumbled using a ceramic mortar and pestle. Plant samples (ca. 200 mg) were solubilized with 3 mL of HNO_3_ and 0.5 mL of H_2_O_2_ (ultrapure reagents) using a Milestone Ethos 900 microwave lab station. The total Pb concentration was quantified by ICP-MS using a Perkin Elmer NexION 350 spectrometer. The analytical quality was evaluated on the basis of the Pb recovery (102%) analyzing the certified standard reference material NCS DC73350 (Leaves of Poplar). Results are expressed on a dry weight basis.

### 2.7. Statistical Analysis

Owing to the limited dataset, to check for the significance of differences in Pb concentrations in soil and lettuce samples amended or not with biochar, a permutation t-test was run using the free software R [30].

## 3. Results

As shown in Table 3, the addition of biochar did not change the soil particle size distribution but significantly increased the pH (from 7.9 to 8.7, on average) and the cation exchange capacity (from 24.8 to 26.3 cmol/kg) of the soil, as well as the pH of the hydroponic solution (from 5.7 to 9.7).

Total Pb concentrations in soils without biochar were in the range 447–860 mg/kg, with a mean value (±standard error) of 562 ± 112 mg/kg. Lead content in the biochar was much smaller (12.4 ± 0.6 mg/kg) and was unable to influence the Pb levels significantly in biochar-amended soils, for which the mean concentration of this element (519 ± 109 mg/kg) was not statistically different (*p* < 0.05) from the untreated soils.

Lead concentrations in the extractable (bioavailable) fraction of soil samples without biochar were in the range of 29.6–76.9 mg/kg, with a mean value of 59.2 ± 11.4 mg/kg (Table 3). The extractable Pb concentrations represented 6.6–9.6% of the total Pb content (8.3%, on average). Soil samples amended with 5% biochar showed a statistically significant reduction of the extractable Pb, in the range of 14–59.5 mg/kg (29.7 ± 5.1 mg/kg), representing 5–8.6% of the total Pb content (6.8%, on average).

Concentrations of Pb in the leaves of lettuce plants grown in the soils amended with biochar (3.7 ± 0.4 mg/kg) were significantly lower (49% less) than levels of the metal in plants cultivated in soils without biochar (7.2 ± 1.7 mg/kg; Table 3). Likewise, lettuce plants cultivated hydroponically with 1% biochar showed a significant decrease (79%) of Pb concentrations compared to those of plants grown in a hydroponic system without biochar (6.3 ± 0.7 vs. 30.2 ± 2.2).

## 4. Discussion

The total Pb concentrations in soils of this study (447–860 mg/kg) were much higher than normal levels of this toxic metal in unpolluted soils (<50 mg/kg), suggesting a strong contamination by tetraethyl-lead spill. As a matter of fact, the world average soil concentration of Pb was estimated to be 17 mg/kg [31], and the median content in topsoils from Europe was 22.6 mg/kg [32]. Additionally, the Italian legislation set two different contamination thresholds for Pb concentrations in the soil, based on land use: 100 mg/kg for green and residential areas and 1000 mg/kg for industrial areas.

The concentrations and percentages of extractable Pb in the soils without the addition of biochar are comparable with the extractable aliquot of the metal in soils with similar Pb contamination levels [33].

As shown in Table 3, the amendment with 5% biochar reduced the extractable fraction of Pb in the soils contaminated by tetraethyl-lead spill by ca. 50%, confirming the capacity of this renewable material to immobilize Pb, as already observed in a long-term experiment [34]. Although the decrease of extractable Pb could be merely linked to a sort of dilution effect occurring as a consequence of the addition of biochar [35], we are confident that the capacity of this biomaterial to increase the soil cation exchange capacity is responsible for the reduction of Pb bioavailability in the amended soils. It is known that this capacity of biochar is mainly due to a number of negatively charged functional groups, such as carboxyl, hydroxyl and amino groups [36,37]. As modeled by Lu et al. [38] using a sludge-derived biochar and suggested by other authors [39], biochar has a large number of surface adsorption sites and exchangeable cations and, therefore, can adsorb Pb in the soil through the following main mechanisms: (1) cation exchange with ions such as Ca^2+^, Mg^2+^, K^+^ and Na^+^; (2) complexation with carboxyl and hydroxyl functional groups; and (3) surface precipitation and/or co-precipitation in poorly soluble compounds (i.e., phosphates).

In this study, the amendment with a biochar characterized by a high cation exchange capacity (96.5 cmol/kg) increased CEC in treated soils (from 24.8 to 26.3 cmol/kg, on average) and consequently the number of functional groups able to adsorb Pb by complexation, thus reducing the potential bioavailability of this metal. In addition, the precipitation of Pb phosphates on the surface of biochar is a further mechanism that can influence the mobility of Pb in the amended soils [40].

It is known that biochar, having a strongly alkaline pH (ca. 10), can also reduce Pb mobility by increasing soil pH [41,42]. In fact, at alkaline pH, the solubility of Pb is greatly reduced [43] and Pb complexes with hydroxyl ions—i.e., the form of Pb least available for uptake by plant roots becomes the dominant form [44]. Measurements performed in our soil culture experiment clearly indicated a significant pH increase (ca. 10%) in soils amended with biochar (from 7.9 to 8.7), confirming that this mechanism is a further possible explanation for the reduced bioavailable Pb found in amended soils.

As a consequence of the reduction in the bioavailable amount, Pb uptake in lettuce leaves was also reduced by ca. 50%. Consistently, an eight-week pot cultivation of *L. sativa* in contaminated soils under five different rice hull biochar amendments (0, 0.5, 1, 2, 5, and 10%) increased soil pH in proportion to the percentage of biochar used and then reduced Pb availability in soils and concentrations in leaves of lettuce plants [45]. Furthermore, a two-week greenhouse experiment with *L. sativa* cultivated in mining soils and amended with two different biochar typologies at two different doses (3% and 7%) showed slight soil pH increases, a notable reduction in the bioavailable Pb fraction from 53% to 91% and a decrease in the Pb accumulation inside the leaves of lettuce in proportion to the amended percentage [46].

The maximum Pb level permitted for leafy vegetables intended for human consumption is 0.3 mg/kg fresh weight [47]. Lettuce plants grown in the contaminated soils of this study had Pb values of 0.36 mg/kg referred to the fresh weight (dry weight was on average 5%), which were thus slightly above the above-mentioned legal limit. However, when 5% biochar was added to the contaminated soils, Pb concentration in lettuce dropped to 0.19 mg/kg—within the legal limit for human consumption.

Compared to lettuce cultivated in the soil, the use of biochar in the plants grown hydroponically caused a greater reduction by ca. 80% in the uptake of Pb, despite a lower amount (1%) of biochar being added. The hydroponic cultivation is a simplified soilless system in which all interfering soil constituents (i.e., clay minerals, Fe oxyhydroxides, organic matter) and microorganisms are omitted [48]. As a consequence, the absence of soil constituents and microorganisms may have favored the efficacy of biochar in limiting the bioavailability of Pb to plants. Our results are consistent with the study by El-Banna et al. [49], who found that oxidized biochar has a great capacity to sorb Pb ions in aqueous solutions. Nevertheless, the readily available nitrate form in which Pb was provided to the hydroponic solutions may have favored the uptake of Pb by lettuce plants. Additionally, the low pH of the solutions without biochar (5.7) may have strongly favored the availability of Pb, while the high pH value of the solutions with biochar (9.7) may have severely limited its bioavailability.

## 5. Conclusions

Soils contaminated by Pb and amended with 5% biochar resulted in a ca. 50% reduction of the extractable (bioavailable) fraction of this toxic metal, limiting by ca. 50% its accumulation in the leaves of lettuce grown in these soils. A similar behavior was found for lettuce plants grown in a simplified soilless system, even with a much higher reduction (ca. 80%) and lower (1%) biochar addition.

The increases in the cation exchange capacity and pH were likely the main factors limiting the bioavailability of Pb in the soil; in addition, complexation with functional groups and precipitation/co-precipitation on the biochar surface and in soil aggregate were the main mechanisms immobilizing this metal.

In conclusion, it is possible to suggest biochar as a useful and renewable biomaterial to allow the cultivation of lettuce in soils contaminated by Pb.

## Figures and Tables

**Table 1:** Characteristics of the biochar used in this study [20]. * = measured in this study.

Particle Diameter (µm)	<500
Nitrogen (%)	<0.5
Potassium (mg/kg)	3020
Phosphorous (mg/kg)	340
Calcium (mg/kg)	9920
Magnesium (mg/kg)	852
Sodium (mg/kg)	291
Total carbon (%)	65%
Water holding capacity (Max, %)	210
pH	9.9
Hash content (%)	7
* Cation exchange capacity (cmol/kg)	96.5 ± 2.2
* Total lead (mg/kg)	12.4 ± 0.6
Recommended dosage (%)	2–10

**Table 2:** Chemical composition of the Atami© B’Cuzz Hydro nutrient solution.

Solution A	Amount
Nitrogen	4.85%
Phosphorous	0.15%
Potassium	4.73%
Sodium	0.19%
Calcium	3.79%
Magnesium	1.32%
Sulphur	0.11%
Iron	0.04%
Boron	0.001%
**Solution B**	**Amount**
Phosphorus	4.1%
Potassium	5.7%
Boron	0.01%
Manganese	0.03%
Molybdenum	0.001%
Zinc	0.039%

**Table 3:** Particle size distribution and mean (±standard error) of pH, cation exchange capacity (CEC) and total and extractable (bioavailable) Pb concentrations in the soil and in leaves of lettuce plants grown in the soil or hydroponically, with and without the addition of biochar. Different letters indicate statistically significant (*p* < 0.05) differences.

	Treatment	
	Without Biochar	With Biochar
Soil particle size distribution		
Sand (%)	13.3	13.8
Silt (%)	82.5	82.0
Clay (%)	4.2	4.2
Texture	silt	silt
Chemical properties		
Soil pH	7.9 ± 0.002 a	8.7 ± 0.005 b
Soil CEC (cmol/kg)	24.8 ± 0.4 a	26.3 ± 0.4 b
Hydroponic solution pH	5.7 ± 0.001 a	9.7 ± 0.007 b
Pb (mg/kg)		
Soil total	562 ± 112	519 ± 109
Soil extractable fraction	59.2 ± 11.4 a	29.7 ± 5.1 b
Lettuce grown in the soil	7.2 ± 1.7 a	3.7 ± 0.4 b
Lettuce grown hydroponically	30.2 ± 2.2 a	6.3 ± 0.7 b

## Data Availability

The raw data presented in this study are available on request from the corresponding author.
